# Supplementary material for: Obesity Risk Assessment for Spanish-Speaking Immigrant Families with Young Children in the United States: Reliability and Validity with Nutrient Values
Source: Children (Basel). 2023 May 12;10(5):868. doi: 10.3390/children10050868 (PMC10217497; doi:10.3390/children10050868)
Supplement: Supplementary file 1 [file children-10-00868-s001.zip › children-2357045-Figures.pdf]

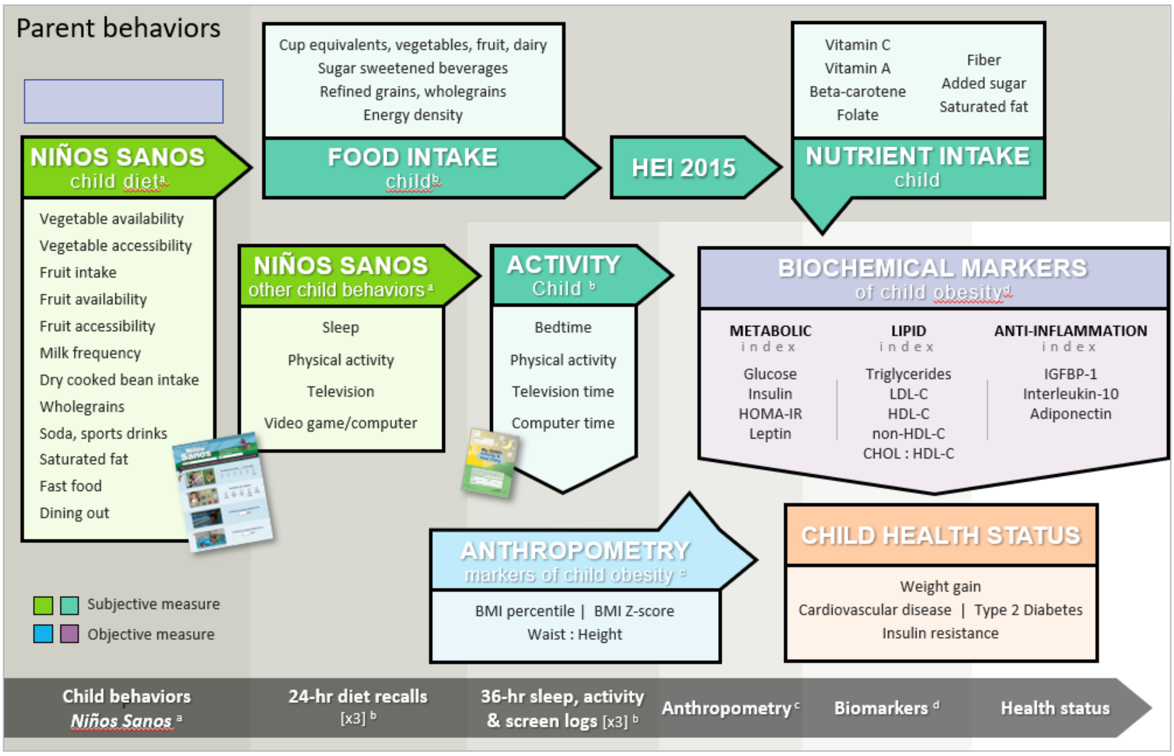

Figure S1. Biopsychosocial framework for validation of Niños Sanos targeting Hispanic families with preschool-aged children.

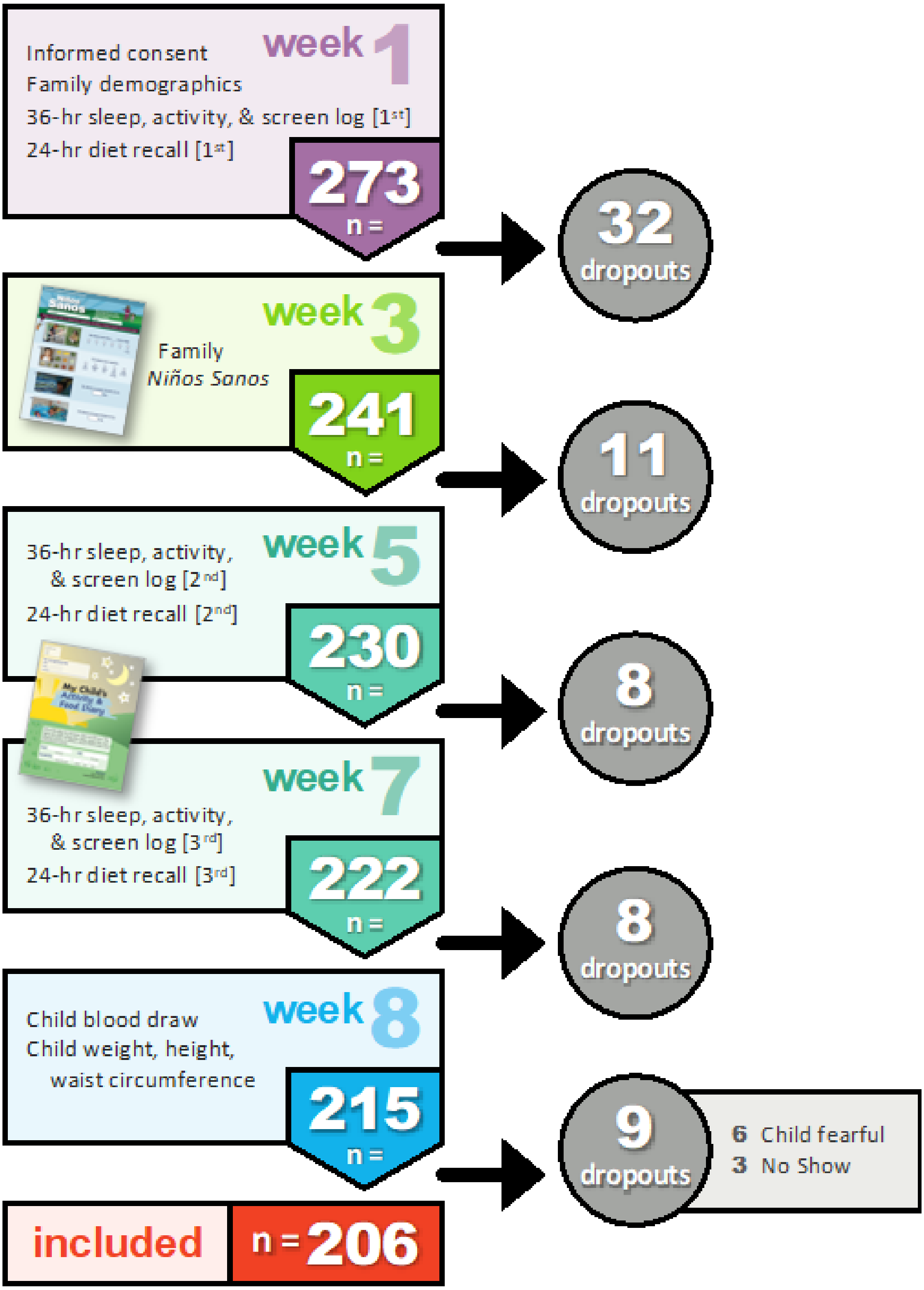

Figure S2. Flow diagram of initial recruits and subsequent dropouts of parent/child dyads at each stage of data collection for the Niños Sanos validation study.
