# Supplementary material for: Obesity Risk Assessment for Spanish-Speaking Immigrant Families with Young Children in the United States: Reliability and Validity with Nutrient Values
Source: Children (Basel). 2023 May 12;10(5):868. doi: 10.3390/children10050868 (PMC10217497; doi:10.3390/children10050868)
Supplement: Supplementary file 1 [file children-10-00868-s001.zip › Supplemental Table S1.pdf]

Supplemental Table S1. Behavioral domain and construct, item text, item visual content and means ± SD for 18 items selected for final version of ***Niños Sanos*** assessment tool.

Behavioral domain & construct	Item text	Item visual	Response[‡] Mean ± SD
**VEGETABLES**			
Vegetable availability	Compro vegetales.<br>I buy vegetables.	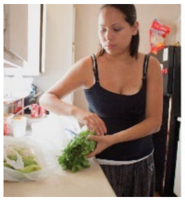 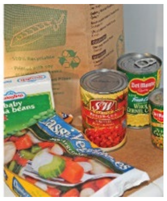	4.2 ± 1.0
Vegetable accessibility	Tengo vegetales listos para que mi niño (a) se los coma.<br>I keep vegetables ready for my child to eat.	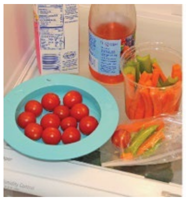 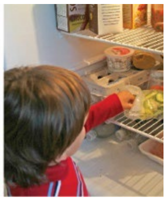	3.2 ± 1.3
**FRUIT**			
Fruit intake	Yo como frutas __ veces al dia.<br>I eat fruit ____times a day.	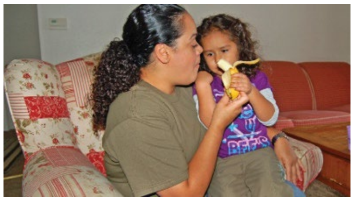	3.3 ± 0.9
Fruit availability	Compro frutas.<br>I buy fruit.	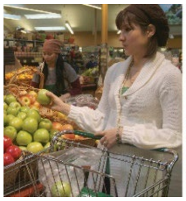 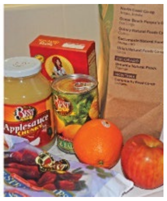	4.4 ± 0.8
**BEANS**			
Dry cooked bean intake	Mi niño (a) come frijoles ___ veces por semana.<br>My child eats beans ____times a week.	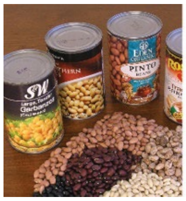 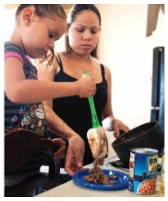	2.4 ± 1.0
**MILK**			

Milk frequency	Mi niño(a) toma leche __ veces al dia.<br>My child drinks milk ___ times a day.	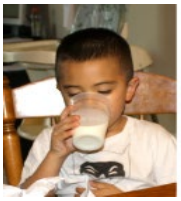 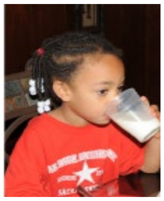	3.3 ± 0.8
Milk frequency	Yo tomo leche __ veces al dia.<br>I drink milk ___ times a day.	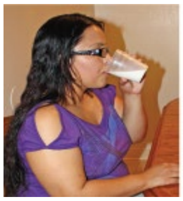 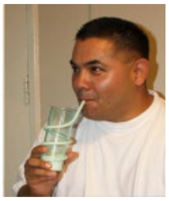	2.3 ± 0.8
**WHOLE GRAINS**			
Milk with cereal	A mi nino(a) le gusta comer cereal en el desayuno.<br>My child enjoys cereal for breakfast.	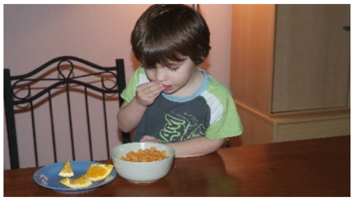	3.0 ± 1.2
**SUGAR SWEETENED BEVERAGES**			
Soda frequency	Mi niño(a) toma sodas __ veces al día.<br>My child drinks soda ____times a day.	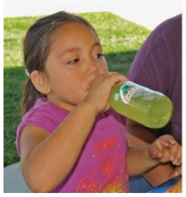 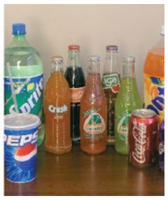	4.7 ± 0.4
Sports drinks, punch frequency	Mi niño(a) toma bebidas deportivas o endulzadas __ veces al día.<br>My child drinks sport drinks or sugared drinks ___ times a day.	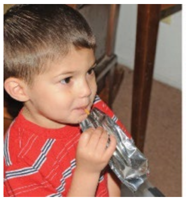 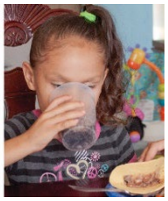	4.5 ± 0.6
**FAT/SATURATED FAT**			
Energy density	Mi niño(a) come comida rápida __ veces a la semana.<br>My child eats fast food _____times a week.	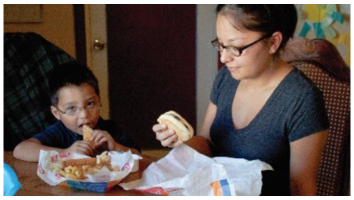	4.4 ± 0.4
Fat, energy density, saturated fat	Le quito la grasa a la carne antes de comerla.<br>I trim fat before eating.	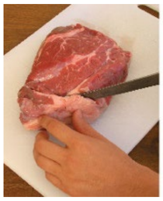 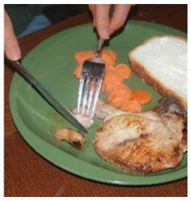	4.3 ± 1.1
**SNACK FOODS**			

Energy dense foods for snack	Mi nino(a) come snacks como papitas (chips), galletas y dulces.<br>My child eats snack foods like cookies, chips and candy.	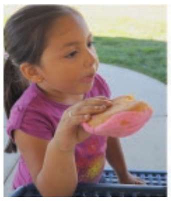 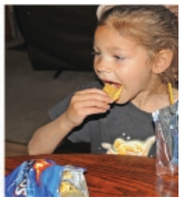	4.0 ± 0.7
**DINING OUT**			
Energy density	Nosotros comemos fuera __ veces a la semana<br>We eat out ____times a week.	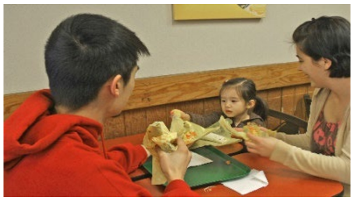	4.1 ± 0.8
**COOKING**			
Energy density	Preparo las comidas para mi niño (a).<br>I cook my child's dinner from scratch.	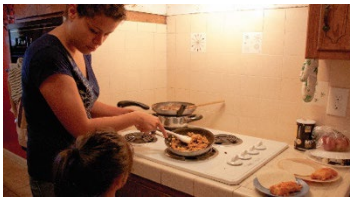	4.6 ± 0.7
**SCREEN TIME**			
Television	Mi niño(a) mira la televisión __ horas al dia.<br>My child watches TV ___ hours a day.	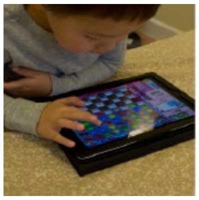 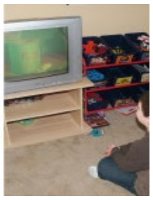	3.7 ± 0.6
**PHYSICAL ACTIVITY**			
Play, sedentary time	A mi niño(a) le gusta jugar en lugar de ver televisión.<br>My child likes playing instead of watching TV.	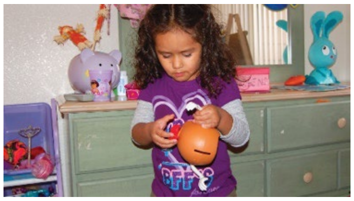	3.4 ± 1.1
**SLEEP**			
Bedtime	Mi niño(a) se acuesta alrededor de las __ PM.<br>My child goes to bed around ___ P.M.	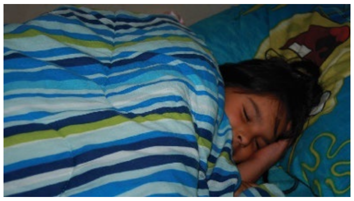	3.0 ± 0.8

‡ Each item has a minimum of 1 and maximum of 5 points; responses as means ± standard deviations.
